# Effective Vote Markets and the Tyranny of Wealth

**DOI:** 10.1007/s11158-017-9371-4

**Published:** 2017-11-13

**Authors:** Alfred Archer, Bart Engelen, Viktor Ivanković

**Affiliations:** 1 Department of Philosophy, Tilburg School of Humanities, Warandelaan 2, 5037 AB Tilburg, The Netherlands; 2 Doctoral School of Political Science, Public Policy and International Relations, Central European University, Nador u. 9, Budapest, 1051 Hungary

**Keywords:** Vote markets, Markets, Limits of markets, Political theory, Exploitation, Voting, Suffrage, Votes, Tyranny of wealth, Vote buying, Vote selling

## Abstract

What limits should there be on the areas of life that are governed by market forces? For many years, no one seriously defended the buying and selling votes for political elections. In recent years, however, this situation has changed, with a number of authors defending the permissibility of vote markets (e.g. Freiman 2014). One popular objection to such markets is that they would lead to a tyranny of wealth, where the poor are politically dominated by the rich. In a recent paper, Taylor (*Res Publica* 23(3):313–328, 2017. doi:10.1007/s11158-016-9327-0) has argued that this objection can be avoided if certain restrictions are placed on vote markets. In this paper we will argue that this attempt to rebut an argument against vote markets is unsuccessful. *Either* vote markets secure their purported benefits but then they inevitably lead to a tyranny of wealth, *or* they are restricted so heavily that they lack the features that have been claimed to make vote markets attractive in the first place. Using Taylor’s proposal as a test case, we make the more general claim that vote markets cannot avoid the tyranny of wealth objection and bring about their supposed benefits at the same time.

## Introduction

What limits should there be on the areas of life that are governed by market forces? This question has received a great deal of attention in recent years with debates about the ethics of for-profit prisons and of markets for bodily organs or surrogacy. Until recently, one issue that remained firmly off the table in these discussions was whether there are good moral reasons to object to markets for votes in political elections. In his discussion of the moral limits of markets, Sandel (1998, pp. 114–115) claimed that ‘No one defends the outright purchase and sale of votes.’ This claim has been echoed more recently by Satz (2010, p. 102).

The few political scientists that did defend the buying and selling of votes at the time Sandel and Satz made these claims, have received more and more backing recently. According to Freiman (2014), we have good reason to lift the prohibition on the buying and selling of votes. Similarly, Brennan and Jaworski argue that ‘Vote selling is not in principle wrong’ (2015, p. 183), and Lippert-Rasmussen has argued that ‘Vote buying is not undemocratic *per se*’ (2011, p. 126).^1^ While there are many objections commonly raised against the commodification of votes, one of the most forceful is that it would lead to the wealthy being able to politically dominate the poor (Archer and Wilson 2014, p. 3; Satz 2010, p. 102; Tobin 1970, p. 269). If votes can be sold and bought at some price, the rich could simply use their money to buy more votes and, hence, more political influence. This would clearly run counter to the egalitarian idea underlying the ‘one-person, one-vote’ principle in contemporary democracies. Allowing vote markets facilitates the translation of economic inequalities, which are often quite large, into political inequalities.

However, this objection has recently been challenged by Taylor (2017c). Taylor is not a defender of vote markets and thinks both that many of the positive arguments in favour of vote markets are unsuccessful (Taylor 2016, 2017a, b; Forthcoming a) and that the burden of proof lies with the defenders of such markets rather than those who oppose them (Taylor Forthcoming b). Nevertheless, he argues that the tyranny of wealth objection is only effective against unrestricted markets in votes and that introducing restrictions to vote markets allows their supporters to overcome this objection. In this paper, we will argue against Taylor’s arguments for the claim that restricted vote markets do not result in a tyranny of wealth. We show how vote markets will either be vulnerable to the tyranny of wealth objection or largely fail to secure the benefits of markets. While Taylor may be right in stressing the possibility of constructing a vote market that does not result in such a tyranny, its restrictions cause it to lack the features that make such markets attractive in the first place.

Our discussion will proceed as follows. In ‘The Benefits of Vote Markets’ we will explain the advantages that have been claimed for vote markets. We will then, in ‘The Tyranny of Wealth Objection’ introduce the tyranny of wealth objection, before explaining Taylor’s response to this objection in ‘Taylor’s Response’. In ‘The Return of the Tyranny of Wealth Objection’ we will argue that Taylor’s response to this objection is unsuccessful. Next, in ‘Restricted Vote Markets Reap Less of the Benefits’ we show that additional restrictions can help avoid the objection but only at the cost of the purported benefits of vote markets. In the ‘Conclusion’ we formulate the more general conclusion that there seems good reason to think that any attempt to restrict vote markets in order to counter the tyranny of wealth objection will be unable to retain the claimed advantages of vote markets.

## The Benefits of Vote Markets

What reasons are there to even consider lifting the legal ban on vote markets? According to Freiman (2014), there are four such reasons. Each is defeasible, and so capable of being overridden by good reasons to the contrary. Nevertheless, Freiman claims that together they offer a presumptive case in favour of legalising vote markets.

The first reason that Freiman (2014, p. 761) offers is that in normal conditions, voluntary economic exchanges are predicted to benefit everyone involved, thus leading to efficiency gains (see also Philipson and James Jr. 1996). People will generally only engage in such exchanges if they judge that doing so will make them better off (Levmore 2000, p. 115). As a result, lifting a ban on vote markets will create legally permissible opportunities for both vote buyers and sellers to make themselves better off.^2^ People can sell their vote if they place more value on the monetary gain than on the political influence their vote gives them. Similarly, others will buy votes if they place more value on the political influence than on the price they have to pay for this. Vote markets thus allow mutually beneficial trades that contemporary democracies are missing out on.

The second reason that Freiman (2014, pp. 762–763) gives is that legalising vote markets seems like a straightforward extension of a plausible principle of voter liberty. As Freiman points out, we already allow voters to exercise their right to vote as they see fit, whether that involves not exercising that right or voting out of self-interest or misguided ideological commitments. While we may object to how these voters use their right to vote, few are willing to claim that the state is entitled to interfere with these voters’ decisions. Given this range of liberty, Freiman (2014, p. 763) claims that the electorate should also be given the liberty to decide whether or not to sell their votes.^3^

Freiman’s third reason (2014, p. 764) in favour of legalising vote markets is that it would lead to election results that better reflect the intensities of voters’ preferences (see also Hasen 2000, p. 1332, and Levmore 2000, pp. 113–114). Those with strong preferences for a candidate can now buy votes in order to increase the chances of their candidate winning. Those with only mild preferences can sell their votes, as their political preference will likely be weaker than their desire to make money by selling their vote. As a result, elections will more accurately reflect the intensities of the preferences of the electorate. Freiman argues that this would be a more democratic outcome and illustrates this with an example. Suppose 50.01% of the electorate have very weak preferences for candidate A while the other 49.99% have very strong preferences for candidate B. In this case, Freiman says that the election of candidate B would be preferable, as it would better reflect the preferences of the electorate. Allowing a market in votes increases the probability of this desirable outcome, thereby avoiding the well-known problem of the tyranny of the majority.^4^

Finally, Freiman (2014, p. 765) argues that there is no moral difference between vote markets and other already existing democratic practices. Take ‘logrolling’: a process in which one legislator (A) agrees to vote in support of another legislator’s (B) legislation in exchange for B agreeing to vote in support of A’s legislation. While the legal status of this practice is unclear, it is a common practice that is rarely seen as deserving of prosecution. Freiman claims that apart from the absence of money transfers, this form of bartering is functionally equivalent to vote selling. Given that there is no moral difference between bartering and trading using money, Freiman claims there is no moral difference between logrolling and vote markets. As long as logrolling is permissible, there thus seems little reason to forbid vote markets.^5^

## The Tyranny of Wealth Objection

Freiman accepts that his case for legalising vote markets is defeasible if sufficiently weighty arguments can be made against it. We will investigate one such argument, according to which allowing vote buying and selling inevitably leads to a tyranny of the wealthy over the poor. This objection is made by Satz (2010, p. 102): ‘A market in votes would have the predictable consequence of giving the rich disproportionate power over others since the poor would be far more likely than the rich to sell their political power.’ Given that the rich have more purchasing power to buy votes and the poor have greater incentives to sell their votes, vote markets will lead to the rich yielding greater political power than the poor. Satz takes this to come in the form of greater representation of political preferences.^6^ We might also think, as Tobin (1970) and Christiano (1990, p. 178) did, that the rich would come to possess greater coercive power over the poor if vote markets were legalised. As Freiman (2014, p. 767) acknowledges, both possibilities are problematic given our (and Freiman’s) acceptance of the liberal commitment to minimising inequalities in political representation. This point has been raised previously by Buchanan and Tullock (1962, p. 271) in the following: ‘If the distribution of *economic* power among the citizens is unequal, open buying and selling of political votes might be said to give “unfair” advantages to the richer members of the group’.

Freiman suggests, however, that absent vote markets, modern democracies are still characterised by inequalities in wealth that cause the rich to yield disproportionate political power. He claims that even ‘without vote markets, better funded, better connected, and better organised groups tend to control the electoral process for their benefit’ (Freiman 2014, p. 767). This suggests that the objection proves too much, and that those concerned with the tyranny of wealth must also admit that current democratic systems lack legitimacy.^7^ In response, we may attempt to alleviate the inequalities in political power by introducing egalitarian regulations to the democratic process as we know it. These can consist in capping the total amount of money that candidates or political parties can spend on campaigns or prohibiting them from spending any private money.^8^

More importantly, Freiman (2014, pp. 768–769) suggests that there is no reason to suspect that a regulated system *with* a vote market is less effective in avoiding the tyranny of wealth than a regulated system *without* a vote market. More specifically, if the regulation consists in equalising background conditions in wealth, then one group would not have an edge over another in its capacity to purchase votes. According to Freiman, regulations in equalising wealth can even do more for democratic systems in the case of vote markets than campaign regulations, because vote markets are not as complex as campaign regulations, and are thus less difficult to oversee.

Archer and Wilson (2014) offer three objections to Freiman’s response. First, Freiman might be making an inappropriate comparison between a regulated system with and without a vote market, as the regulations themselves might implicate a prohibition on vote markets (2014, p. 2). Second, if we are to choose between two democratic regimes producing similar degrees of inequalities, Archer and Wilson (2014, pp. 4–5) argue, the one with a vote market may be less permissible due to other grounds of legitimacy. For instance, we may think that power inequalities are often derived from unregulated private control over media outlets, but we may allow for those inequalities if prohibiting them would effectively diminish free speech. Freiman would have to convince us that prohibiting vote markets similarly endangers some fundamental value, and hence that the inequality in power it produces is permissible all-things-considered.^9^ Third, Archer and Wilson suggest that, while some regulations are available to tackle inequalities in power arising from wealth in current political systems, the same cannot be said about the inequalities arising in vote markets. Imagine a system in which each citizen is provided by the government with an equal sum she can spend on buying the votes of others. This would not be sufficient in neutralising the power differential because the poor would still have a bigger incentive to sell than the rich (Archer and Wilson 2014, p. 4).

Some might point to a general worry that wealth enables the rich to dominate the poor in electoral systems regardless of whether vote markets are implemented. The suggestion is that this is achieved either through direct or, if regulatory obstacles are in place, indirect ways of spending, maintaining the stranglehold of the rich over the poor. But if the tyranny of the rich is inevitable in contemporary democracies, then the concern of tyranny brought about by vote markets might not be looming large over our heads.^10^

However, even if it is the case that the rich will be able to tyrannise the poor in the absence of a vote market, there remain three good reasons to establish the ethical desirability of vote markets (or a lack of one). First, we might think unrestricted vote markets alone have the capacity of bringing about the tyranny of wealth, as we indeed claim here. If contemporary democracies without vote markets are indeed plagued by a tyranny of wealth in other ways, but are eventually able to eliminate the tyranny in the future, the dangers of the tyranny re-emerging should be found in unrestricted vote markets. Second, establishing the moral character of unrestricted vote markets can give us reasons regarding why certain practices, such as earmarking and logrolling, are impermissible, if we can show they are in relevant ways analogous to unrestricted vote markets. Third, tyranny of wealth might be a matter of degree. If that is the case, unrestricted vote markets might only be bolstering tyrannies, but not causing them to emerge. Taking this approach still gives us good reason to oppose unrestricted vote markets if they indeed bolster a tyranny of wealth, as it should be in our moral interest to alleviate tyranny.

## Taylor’s Response

Taylor agrees that the tyranny of wealth objection to vote markets should be taken seriously as long as it entails that a) the poor would be the likely sellers of votes and b) the rich the likely buyers (Taylor 2017c, p. 321). With a fixed price set for a single vote, the poor clearly have a greater incentive to sell their votes, since the money received from the transaction, relative to their total wealth, has greater value to them than to the rich. The rich, on the other hand, are the more likely buyers because the transaction bears smaller costs to them, relative to the money they can spend, than to the poor.^11^

According to Taylor, vote market advocates should first even up the incentives for members of different social classes. In Taylor’s example, we are to assume that there are three classes in society—the poor, the middle class, and the rich—with differing levels of total median wealth. The incentive of a poor voter (with a total median wealth of $10,000) to sell her vote for $1 can then be assumed to be equal to that of a middle-class voter (with $500,000) to sell her vote for $50 or that of a rich voter (with $100,000,000) to sell her vote for $10,000 (Taylor 2017c, pp. 323–324). Call this Taylor’s *Proportionality Condition*: in vote market transactions, the prices of each individual’s vote are to be set proportionally to each voter’s wealth.^12^

While dictating prices proportionally to wealth settles the problem of the disproportionate incentive to sell, it does little for resolving the problems of the rich being the likely buyers and the poor being the likely sellers. As the rich still have the most purchasing power, they would simply turn their attention to the affordable price of poor votes. Thus, Taylor suggests a further restriction to vote markets, which we call the *Package Condition*: in order to purchase poor votes, a buyer is required, relative to group numbers, to buy some votes from other social classes. In Taylor’s imagined society with 30 poor, 60 middle-class and 10 rich people, a vote package consists of three poor votes, six middle-class votes and one rich vote, while votes in excess would be disqualified (Taylor 2017c, pp. 324–325). This restriction eliminates the likely seller problem. However, as Taylor rightly points out, the likely buyer problem remains. The rich would still manage much greater resources and would be more likely to buy vote packages.

To solve this problem, Taylor instructs, imagine a society with two parties, the Poor Party and the Rich Party, where two classes, the poor majority and the rich minority, predictably support their parties along class lines. Given that a single vote is not likely to change the outcome of the election, the members of the poor would have an incentive to quickly sell off their vote before other poor voters do the same, with hopes of not compromising a good outcome for their party. If a sufficient number of poor voters defect, the rich would still be able to change the outcome of the election (Taylor 2017c, p. 325). According to Taylor (2017c, pp. 326–327; see also Levmore 2000, p. 123), the poor face bigger obstacles in coordinating their efforts (forming a ‘voter cartel’ and agreeing to vote for the Poor Party) than the rich (forming a ‘vote-*buying* cartel’). Given the poor’s numbers and their lack of resources, they will not be able to overcome the collective action problem.

To solve this, Taylor adds a final restriction. The *Competition Condition* stipulates that there are at least two competitors with equal budgets to buy off votes. This ensures that vote sellers are motivated by something other than monetary incentives. Since vote sellers are guaranteed the same amount of money whichever party they choose to sell their vote to, non-monetary incentives will play a part in their decision: ‘while it would still be rational for the poor to sell their votes the prospective buyers would still have to engage in “[a] competitive struggle for [some of] the people’s vote[s]” on the grounds of something other than price’ (Taylor 2017c, p. 327). Interestingly, Taylor moves over several issues rather quickly here. While he frames this as one further and final restriction, it in practice includes (at least) two: a *Competition Condition* and an *Equal Budget Condition* (which stipulates that all potential buyers manage ‘equivalent vote-buying budgets’).^13^

In this section we have explained Taylor’s defence of vote markets against the tyranny of wealth objection. According to Taylor, this objection can be avoided by restricting vote markets so that they conform to the *Proportionality, Package, Competition* and *Equal Budget Conditions.*

## The Return of the Tyranny of Wealth Objection

Let us start by pointing to several problematic aspects of Taylor’s proposal. Firstly, according to Taylor’s *Proportionality Condition*, one’s vote price should be set proportionally to the median wealth of one’s economic class. This has the strange and objectionable result that the vote market scheme is *de facto* making the rich even richer. A rich vote is worth $10,000, which equals the total median wealth of a member of the poor class. So while the standard objection against vote commodification is that it translates economic inequalities (in wealth) into political inequalities (in power), Taylor’s proposal translates political equality (one-person, one-vote) into economic inequalities! While this aspect in itself does not so much constitute a tyranny of wealth, since it does not enable the rich to politically dominate the poor, it does constitute a substantial bonus for the wealthy.

In order to see how the tyranny of wealth objection re-emerges, let us focus on Taylor’s imagined society (2017c, pp. 324–325) with 30 poor, 60 middle class and 10 rich people. Here, a vote package costs $10,303.^14^ One can easily see how this proposal does not live up to its egalitarian promises, since such a package only costs $303 more than the net value of one rich person’s vote while exceeding the total wealth of a poor person. The only buyers of vote packages will thus be the rich (or a Poor Party that pools the poor’s resources). Since no single poor person is able to buy a vote package, they can only enter into the vote market as sellers and therefore miss out on some of the vote market’s benefits. In contrast, the rich can both buy vote packages (still relatively cheaply to them) and sell their votes (in which case they make more money than the poor do).

One can illustrate this problem in another way. If everyone were willing to sell her vote, a single rich person could buy up all votes, which together would cost him $103,030 (around 0.1% of her total wealth). Conversely, one would need a group of 11 poor people spending nearly all their wealth to acquire the opportunity for such political influence. The tyranny of wealth objection clearly returns in full force.

To make matters worse, the *Package Condition* can lead to package prices that can turn out to be even more harmful to the poor. With a different ratio between poor, middle class and rich, say 4:3:3, a vote package could cost $30,154, further narrowing the range of prospective buyers to the rich. Another implementation problem arises as well. If we were to set class boundaries differently and add more classes, this would result in a larger single vote package and higher prices. Next to the arbitrary character of all this, it in effect imposes further obstacles for the poor to buy votes.

Another problem with Taylor’s proposal is that to avoid the return of the tyranny of wealth objection, Taylor needs to add more restrictions. One is that private citizens or companies are not allowed to enter the vote market as buyers. Such a *Political Parties Only Condition* is needed to avoid the scenario where the rich can easily outbid the poor (even if the latter coordinate efforts) (Levmore 2000, pp. 135–136). The restriction to political parties, understood as citizens forming political platforms, however, is not enough. After all, the rich can create such platforms much more easily than the poor.

The *Equal Budget Condition* does not help much here. If all 90 poor and middle-class people would decide to organise politically and invest half their wealth, it would only take three rich people investing about half their wealth to match that vote buying budget ($151,500,000). So if political parties budgets should be equal but also substantial, then they can be formed by relatively large donations of almost all poor people (call this the ‘Bernie strategy’), by a few large donations from the relatively rich (the ‘Hillary strategy’), or by one huge donation from a really rich person (the ‘Donald strategy’).

Taylor (2017c, p. 327) accepts that, under these conditions, the rich are the likely vote buyers: ‘the rich could still politically dominate the poor through disproportionately participating in the market as buyers.’ However, he claims that this is not as problematic as we might think. With the *Competition* and *Equal Budget Conditions* in place, prospective buyers will engage in a competitive struggle for people’s votes ‘on the grounds of something other than price […]. In such a situation the parties would compete for the votes of those of the poor who had at least a minimal interest in the results of the election on policy grounds’ (Taylor 2017c, p. 327). Given that these parties have to provide some non-monetary reason for the poor to sell them their votes, they have to appeal to the political views of the poor, which are thus guaranteed political representation.

However, this solution is not enough to stave off the tyranny of wealth objection. Consider a political system in which all of the conditions suggested so far are met: votes are bought in packages (*Package Condition*), vote prices are set proportional to people’s wealth (*Proportionality Condition*), there is more than one party that can buy votes (*Competition Condition*) and potential buyers have equal budgets (*Equal Budget Condition*). Now assume that two political parties represent competing interests of the rich (call them Conservatives and Liberals) and one party represents the interests of the poor (call them Socialists). Suppose that both the Conservatives and the Liberals have a large amount of money to buy vote packages but the Socialists do not. In fact, this scenario is more than a mere theoretical possibility, as it is the parties that represent the interests of the rich that would be most likely to attract wealthy donors. Again, the poor would likely be politically dominated by the rich here, since the poor who value the money more than their vote can only sell to the Conservatives or the Liberals. Such a vote market therefore incentivises poor sellers, in contrast to rich sellers, to go against their interests. Again, the interests of the rich are likely to politically dominate the poor, which would constitute a tyranny of wealth.^15^

The problem here is that not all political parties standing for election are able to enter the vote market, which could be avoided by introducing yet another restriction on the vote market. Call this the *All Parties Are Buyers Condition*: all parties competing in the election must be potential vote buyers. The problem with this move though is that it places high entry costs on any political party hoping to stand for election. With the *Equal Budget Condition* in place as well, the Socialists can now only stand for election if they can match the funding of the other parties. If only parties with sufficient resources have a reasonable chance of getting votes, most of these will have to be supported by wealthy donors. As a result, this situation favours parties with wealthy voters (the ‘Hillary’ and ‘Donald strategy’) and so predictably leads to the interests of the rich dominating the interests of the poor (since the ‘Bernie strategy’ has trouble overcoming the high entry costs).

In this section we have argued that even a vote market that possesses the constraints that Taylor proposes would still be vulnerable to the tyranny of wealth objection. Taylor’s initial restrictions (the *Package*, *Proportionality* and *Competition Conditions)* are insufficient to avoid a situation where the political preferences of the rich dominate those of the poor, because the parties representing the latter experience trouble entering the vote market as buyers. While further restrictions (the *Equal Budget* and *Political Parties Only Conditions*) can ensure that all parties are capable of buying votes, they introduce high entry costs, which again predictably lead to an exclusion of the poor’s political preferences. In sum, Taylor’s proposal does not succeed in showing that a regulated vote market system can solve the problem of wealth-based power inequalities better than a regulated system without a vote market, as Freiman (2014, pp. 768–769) suggested.

## Restricted Vote Markets Reap Less of the Benefits

The previous section shows that vote markets, even with Taylor’s constraints in place, fail to adequately address the tyranny of wealth objection. In this section, we show how this problem can be fixed by introducing additional restrictions. While Taylor may thus be right in claiming that not all vote markets are vulnerable to the tyranny of wealth objection, we will argue that the avalanche of required restrictions will inhibit such vote markets from reaping their purported benefits. It is worth noting again that Taylor has argued in a series of papers that the purported benefits of vote markets are illusory (Taylor 2016, 2017a, b; Forthcoming a). It seems reasonable to assume, however, that not all supporters of vote markets will be fully persuaded by all of Taylor’s arguments (such is the nature of philosophical debates). Our aim is to show that avoiding the tyranny of wealth objection by introducing additional restrictions to vote markets will make it even harder to make the case that vote markets would bring about any of these purported benefits.

The high entry costs that potential vote buyers face and that benefit Rich over Poor Parties can be avoided by, for example, capping the amount of money that any private person or company can donate to political parties (a *Restricted Donations Condition*) or capping the party budgets themselves (a *Restricted Budget Condition*). Taylor (2017c, p. 327) himself briefly hints at the latter: ‘Restricting how much could be spent on the direct purchase of votes to ensure that at least two prospective vote buyers would be equivalently capitalised would thus protect the poor from disenfranchisement.’

While these fixes would indeed avoid a tyranny of wealth re-emerging, they also raise new questions. If donations are restricted, where is the funding for political parties to come from? And how low should the cap on budgets be for the less affluent political parties to access the market as well? The lower the cap, the narrower the scope of the vote market, since political parties will be able to buy fewer vote packages.

Of course neither of these problems is decisive. Political parties could be funded by the state and it may be accepted that vote markets are limited. We return to both possibilities later in the paper. The real issue we want to stress here is that the proposal we end up with no longer succeeds in generating the benefits that vote markets have and that provided the reasons to consider vote markets in the first place. In what follows, we examine each of the three benefits Freiman (2014) mentions: reflecting the intensity of preferences, generating mutually beneficial results and respecting and expanding voter liberty.^16^

The first purported advantage Freiman refers to is that vote markets bring about election results that better reflect the intensity of the electorate’s preferences. People with strong preferences can buy the votes of those with weaker preferences, at a price that reflects the strength of both sets of preferences. While unrestricted vote markets can achieve this (Levmore 2000), a Taylor-style restricted vote market does so to a far lesser extent.

First, the *Proportionality Condition* implies that people can only sell their vote for a fixed price. This would not reveal the preference intensity of sellers as effectively as auctioning off votes would (Philipson and James Jr. 1996, p. 252). The only thing we come to know about the ‘supply side’ of a Taylor-type vote market is whether people value their vote more or less than a specific amount of money. This is roughly similar to an electoral system without a vote market, where people with intense political preferences are more likely to cast their vote (and organise politically in other ways such as rallying or forming interest groups) than those without intense preferences, who are more likely to abstain (and refrain from other kinds of political action). Also, there are several voting methods that allow voters to express preference intensity, such as the Borda count (García-Lapresta and Martínez-Panero 2002). Compared to these, restricted vote markets do not better reflect preference intensities at all.

In response, Taylor could point out that his proposal provides *at least some* information about the intensity of preferences across the electorate.^17^ While this may be true if we consider Taylor’s original proposal, which remained vulnerable to the tyranny of wealth objection, we need to investigate whether this also holds in a vote market with the further restrictions in place that are needed to prevent the tyranny of wealth.

If the *Political Parties Only Condition* is met, the electorate will no longer be able to buy votes. This hardly gives a better reflection of the intensity of preferences at the ‘demand side’ of the vote market. If a rich person cannot buy votes, this restricts his opportunity to express how intensely he prefers some electoral outcome. More problematic in egalitarian terms is the scenario where the poor have a hard time forming a political party that is able to buy vote packages. If they fail to do so, the poor cannot reap the benefits that the vote market supposedly brings to buyers.

The *Restricted Donations* and *Restricted Budget Conditions* also limit the extent to which people with intense preferences can satisfy these on the market. With a low cap on private donations, the playing field is indeed levelled but only a limited amount of vote packages can be sold and bought. Again, the potential benefits of vote markets will not be achieved to the fullest since many who want to sell their votes may find themselves unable to do so.

The second purported advantage of vote markets Freiman refers to is their ability to generate mutually beneficial exchanges. It seems reasonable to think that Taylor’s proposal would allow for such efficiency gains. After all, anyone who decides to sell votes or organise politically to buy vote packages would presumably only do so because they judge that it would make them better off. However, the relevant question for our purposes is not whether mutually beneficial exchanges would occur if Taylor’s vote market proposal were implemented. Rather, it is whether introducing such a vote market would make people better off. When we keep in mind the constraints imposed by the *Package, Competition* and *Equal Budget Conditions*, it is hard to see how it would, for example, make political parties better off. They can only buy votes if 1) these come from a cross section of classes, 2) there is competition from other vote buyers and 3) their competitors have an equal budget for votes. According to Taylor, this ensures that voters make decisions about whom to sell their vote based on political preferences rather than financial incentives. However, if this is the case, then political parties would be paying for votes that they would also have received if there had been no vote market in place.^18^ This seems to make them clearly worse off, not better off.

In response to this argument it might be claimed that at least some of the votes that political parties buy are ones that they would not have received otherwise: the votes from the politically apathetic who would have abstained in the absence of a vote market. So parties buying votes *are* benefitting from the vote market as they collect votes they would otherwise not attract. However, this benefit is illusory since their competitors will manage to buy such votes as well. So while such a vote market will lead to an increase in votes being cast, the vote buyers will not be benefitted overall.

There are other reasons why Taylor-style vote markets would fail to bring about efficiency gains and thus fail to benefit all (or at least most). Remember how Taylor’s *Package Condition* implied that vote prices were not governed on the basis of each person’s willingness to pay and to accept. Compared to a perfectly competitive vote market, where vote prices are shaped by the forces of supply and demand, Taylor’s proposal with fixed prices will not maximise the efficiency gains (in terms of consumer and producer surpluses) and there will be deadweight losses. In fact, there are efficiency arguments against vote buying, a practice that involves money being spent merely on gaining political advantage over political adversaries. This kind of ‘rent seeking’ Hasen (2000, pp. 1332–1333) rightly argues, is inefficient because the money spent in vote markets cannot be put to proper social use anymore. Downs (1957, p. 192) made a similar point, claiming that vote markets will not lead to Pareto-superior outcomes, since they create negative externalities: ‘transactions therein will almost inevitably make someone worse off’. These and other problems (see also Philipson and James Jr. 1996, p. 247) made John Ferejohn (1974, p. 25) conclude that, when it comes to vote trading, ‘we don’t know if it has any desirable normative or efficiency properties’.

The third purported advantage of vote markets is that they would increase voter liberty. At first sight, Taylor’s vote market proposal seems to at least retain this advantage. While his restrictions inhibit voter liberty compared to an unrestricted vote market, they still expand people’s choice options—adding the option to sell their vote—compared to a system without vote markets.

Again, however, the situation becomes more complicated when we include the restrictions needed to avoid the re-emergence of the tyranny of wealth. After all, the *All Parties are Buyers Condition* creates high entry costs that allows only those parties with significant resources to compete. To fix this, the *Restricted Donation Condition* needs to be introduced, but this raises the question of where the funding for political parties is to come from. If the state were to fund political parties, this would no longer constitute an increase in voter liberty, as taxpayers would be forced to contribute funds towards political parties so that the latter can purchase votes. The added liberty for some people, who now have the option to sell their vote, does not compensate for this decrease in liberty across the electorate. The other way to avoid significant entry costs to the political process was the *Restricted Budget Condition*. While not forcing taxpayers to fund vote buyers, it does so at the cost of significantly limiting the scope of these vote markets. Again, any increase in voter liberty is doomed to be small.

## Conclusion

Let us conclude by bringing together the two lines of argumentation that we have pursued. In general, we want to claim that vote market proposals face the following dilemma. *Either* they secure the benefits of (vote) markets but then they inevitably lead to a tyranny of wealth, *or* they are restricted so heavily that they fail to secure the benefits that make up their justification. Our general claim is thus that, the better a vote market succeeds in avoiding the tyranny of wealth objection, the less of the supposed advantages of vote markets it will have.

Some of our criticisms of Taylor may be picking holes in the details of a proposal that is not fully developed. Still, we believe it is a good test case for our more general claim that proposals along these lines will inevitably fail to avoid the tyranny of wealth objection (if they are not restricted accordingly) or fail to reap the benefits vote markets are to bring about (it they are restricted accordingly). While proponents of vote markets may try to come up with different proposals, we believe the burden of proof lies squarely with them to show that such markets can both function as proper markets and still avoid the tyranny of wealth.

In our view, the restrictions needed to make vote markets compatible with the democratic principle of political equality are so strict that the scheme will be impossible to implement in practice. In fact, the implementation problems arise from the following tensions between the restrictions themselves:

1. A tension between the two restrictions needed to avoid the likely sellers problem: while the *Proportionality Condition* requires making vote prices proportional to each and every individual’s total wealth (if this could ever be done), the *Package Condition* only works with a limited set of classes (with median total wealth amounts that will be so rough as to re-introduce unequal incentives to sell).
2. A tension between the conditions stipulated and the very idea of (vote) markets: can one really speak of a proper market if individuals can only sell the commodity at hand when circumstances are exactly right (supply side) and when no individual is allowed to buy the commodity at hand (demand side). After all, this is what the *Competition*, *Equal Budget* and *Political Parties Only Conditions* lead up to.

In the end, what Taylor’s proposal amounts to is a monetised version of what representative democracies are already like: a system in which several political parties compete with each other, on an equal footing, for votes that are equally distributed amongst citizens, none of whom is seduced to give their right to vote away for politically irrelevant reasons. This offers no improvement over the kind of legislation and social practices—surrounding campaign funding, television screen time, etc.—in place in contemporary democracies all over the world.

It may well be true then that some heavily restricted vote market could avoid the tyranny of wealth objection. However, the restrictions needed in this respect would be ineffective in bringing about the supposed benefits of vote markets. Vote markets can either avoid the tyranny of wealth objection or they can be effective at bringing about the supposed benefits of vote markets. However, they cannot do both at the same time.

## Footnotes

1 See also Kochin and Kochin’s (1998) defence of the claim that in some circumstances buying and selling votes is unproblematic and Brennan’s (2011, p. 135) defence of vote buying provided that any votes bought are used to promote the common good.

2 We might worry, as George Mankiw (2007) does, that despite benefitting the buyers and sellers, interactions on vote markets may impose costs on third parties. However, as Freiman (2014, p. 762) points out, this argument proves too much, as persuading someone to vote a certain way could also bring about negative consequences for third parties, yet we would not take this to be a reason to prohibit acts of political persuasion.

3 See Taylor (Forthcoming a) for a response to this argument.

4 Posner and Weyl (2015) make a similar argument for a different system of ‘quadratic voting’, where everyone can buy as many votes as she wants from a monopolist seller, with the price being the square of the number of votes she casts. Note that in most contemporary voting systems, at least those without compulsory turnout, B is likely to win as well, given the fact that abstention will be higher amongst the likely voters for A.

5 Freiman (2014, p. 765) makes a similar claim about ‘earmarking’: the practice of promising to set aside public money for specific projects with the aim of gaining the support of parts of the electorate. For a response to Freiman’s arguments concerning both logrolling and earmarking, see Taylor (2016). Also, for an account of different activities similar to vote selling at nominating caucuses and conventions in the US, but not prosecuted by federal law, see Holzer (2008).

6 Saul Levmore argues that a crucial problem with vote markets is that they generate perverse results, undermining the legitimacy and purpose of democratic decision-procedures themselves, because they ‘create situations in which participants will see that the result of an election […] is contrary to the one a majority of their number wished’ (Levmore 2000, p. 152).

7 Some authors may take this route, or at least claim that there is an inherent tension between capitalist markets and democracies. For example, David Copp claims that democratic reasons against economic inequality arising in capitalism are of the same kind as those against vote markets—both undermine a cooperative system in which members hold equal stakes (Copp 2000, pp. 90–91).

8 These and other limits on the role money can play in campaigns or elections have been implemented in most democracies. See, for example, Fisher (2002) for an analysis of the regulations in the United Kingdom since 2000.

9 Interestingly, the case against regulating campaign spending is often made on the basis that this would constitute an unjustifiable restriction on freedom of speech. In one decision, the US Supreme Court decided that putting limits on the amount a person may contribute to all federal candidates and parties combined in a two-year timespan is ‘unconstitutional under the First Amendment’ (Supreme Court of the United States 2014: 1).

10 We thank an anonymous reviewer for raising this point.

11 This holds regardless of the content of the political preferences of rich and poor, which may be self-interested or not. Whether one is a likely buyer or seller depends on the trade-off one makes between the price of votes and the value one attaches to them. One of the many reasons one values votes may be as a means to promote one’s self-interest (‘pocketbook voting’) but this self-interested voter hypothesis has hardly found empirical support (e.g. Feldman 1982).

12 To reach the point at which incentives to sell one’s vote are equalised, Taylor should argue that prices are set relative to each and every specific voter’s total wealth. However, this is going to be extremely hard to implement, given the difficulty of measuring people’s total wealth (which consists of an aggregation of uncertain and constantly changing assets such as incomes, stocks, housing properties, etc.) (Kopczuk 2015) and given the fact that people can have zero or even negative accumulated wealth (when they are in debt). To make this work, vote prices can be made proportional to the median total wealth of that voter’s economic class. This, of course, raises new difficulties with respect to implementation. How many classes should we distinguish? How should we set class boundaries and vote prices? Without going into detail, it suffices to say that this proposal inevitably fails to equalise incentives to sell. When you have two middle-class people, one with $100,000 and one with $1,000,000, the first clearly has more incentive to sell her vote for $50.

13 A third condition might have been implied by Taylor. Since he assumes that political parties are the relevant competitors for vote sellers, it might be that only parties can become the vote-buying cartels Taylor has in mind. More on the *Political Parties Only Condition* in the following section.

14 A package of ten votes is the smallest possible package one could purchase, since the number of votes bought have to match the 3:6:1 ratio of the poor, middle class and rich economic class.

15 Moreover, in order to get round the constraint of the *Equal Budget Condition,* a political party can simply split itself into two parties with nearly identical platforms in order to ensure that they can still buy votes. However, whether these strategies are pursued would depend on the institutional regulations permitting or prohibiting post-election coalitions. We do not go into this complication here.

16 Freiman’s fourth argument for legalising vote markets, that it is equivalent to other democratic practices that are thought of as permissible, is not a claimed benefit of vote markets.

17 In fact, Taylor has rebuked elsewhere the claim that vote markets lead to outcomes that reflect preference intensities better than non-market systems: ‘instead of better reflecting voters’ weighted preferences, vote buying will lead to electoral outcomes that reflect them less accurately’ (Taylor 2017a, p. 107) It is worth noting though, that Taylor’s defence of vote markets against the tyranny of wealth objection (Taylor 2017c) can be accepted independently of Taylor’s argument that vote markets would not better reflect voter preferences (Taylor 2017a). Our arguments then should persuade those who believe (1) that vote markets do more accurately reflect preference intensities (and thus go against Taylor 2017a) and (2) that vote markets can avoid the tyranny of wealth objection at the same time (in line with Taylor 2017c).

18 One could object by stressing that voters are not obliged to sell their vote: they can decide to keep them and cast them themselves. However, on the basis of Taylor’s assumption that sellers will be making their decisions based on political judgements, his ideal scenario is that citizens do sell their vote to whatever party that they would have voted for otherwise. This way, after all, citizens can cash in and register their political preference.
